# Chemical Inhibition of Bromodomain Proteins in Insect-Stage African Trypanosomes Perturbs Silencing of the Variant Surface Glycoprotein Repertoire and Results in Widespread Changes in the Transcriptome

**DOI:** 10.1128/spectrum.00147-23

**Published:** 2023-04-25

**Authors:** Ethan C. Ashby, Jennifer L. Havens, Lindsey M. Rollosson, Johanna Hardin, Danae Schulz

**Affiliations:** a Department of Biology, Harvey Mudd College, Claremont, California, USA; b Department of Mathematics and Statistics, Pomona College, Claremont, California, USA; Johns Hopkins University Bloomberg School of Public Health

**Keywords:** bromodomain proteins, epigenetics, gene regulation, molecular parasitology, parasite, *Trypanosoma brucei*

## Abstract

The eukaryotic protozoan parasite Trypanosoma brucei is transmitted by the tsetse fly to both humans and animals, where it causes a fatal disease called African trypanosomiasis. While the parasite lacks canonical DNA sequence-specific transcription factors, it does possess histones, histone modifications, and proteins that write, erase, and read histone marks. Chemical inhibition of chromatin-interacting bromodomain proteins has previously been shown to perturb bloodstream specific trypanosome processes, including silencing of the variant surface glycoprotein (VSG) genes and immune evasion. Transcriptomic changes that occur in bromodomain-inhibited bloodstream parasites mirror many of the changes that occur as parasites developmentally progress from the bloodstream to the insect stage. We performed transcriptome sequencing (RNA-seq) time courses to determine the effects of chemical bromodomain inhibition in insect-stage parasites using the compound I-BET151. We found that treatment with I-BET151 causes large changes in the transcriptome of insect-stage parasites and also perturbs silencing of VSG genes. The transcriptomes of bromodomain-inhibited parasites share some features with early metacyclic-stage parasites in the fly salivary gland, implicating bromodomain proteins as important for regulating transcript levels for developmentally relevant genes. However, the downregulation of surface procyclin protein that typically accompanies developmental progression is absent in bromodomain-inhibited insect-stage parasites. We conclude that chemical modulation of bromodomain proteins causes widespread transcriptomic changes in multiple trypanosome life cycle stages. Understanding the gene-regulatory processes that facilitate transcriptome remodeling in this highly diverged eukaryote may shed light on how these mechanisms evolved.

**IMPORTANCE** The disease African trypanosomiasis imposes a severe human and economic burden for communities in sub-Saharan Africa. The parasite that causes the disease is transmitted to the bloodstream of a human or ungulate via the tsetse fly. Because the environments of the fly and the bloodstream differ, the parasite modulates the expression of its genes to accommodate two different lifestyles in these disparate niches. Perturbation of bromodomain proteins that interact with histone proteins around which DNA is wrapped (chromatin) causes profound changes in gene expression in bloodstream-stage parasites. This paper reports that gene expression is also affected by chemical bromodomain inhibition in insect-stage parasites but that the genes affected differ depending on life cycle stage. Because trypanosomes diverged early from model eukaryotes, an understanding of how trypanosomes regulate gene expression may lend insight into how gene-regulatory mechanisms evolved. This could also be leveraged to generate new therapeutic strategies.

## INTRODUCTION

The African trypanosome Trypanosoma brucei, the causative agent of human and animal African trypanosomiasis, cycles between a mammalian host and a tsetse fly vector. Large differences in the two host environments necessitate extensive adaptation on the part of the parasite to ensure survival. This adaptation is facilitated by large changes in the transcriptome of parasites within each host (1–6). Parasites living in the bloodstream vary the proteins on their surface using a large repertoire of variant surface glycoprotein (VSG) genes (7) in order to evade the host immune system. Following transition to the fly after a blood meal, parasites remodel their surface proteins to express an invariant procyclin protein coded for by the procyclin rich in Glu-Pro repeats (EP) and procyclin rich in Gly-Pro-Glu-Glu-Thr repeats (GPEET) gene family (8) and alter their metabolism to adapt to a glucose-poor environment (9, 10). Within the fly, the parasites migrate from the midgut to the salivary gland, passing through a number of developmental stages before remodeling their surface proteins once more to prepare for entry into the mammalian bloodstream. The surface proteins expressed by these salivary gland metacyclic parasites are termed metacyclic VSGs (mVSGs) (11, 12). Because trypanosomes transition through a number of life cycle stages and diverged quite early compared to better-studied model organisms, they serve as a model for gene-regulatory mechanisms that facilitate transcriptome reprogramming and adaptation in early-branching eukaryotes.

Trypanosomes have a number of unusual gene-regulatory features, including polycistronic gene transcription and trans-splicing and polyadenylation of mRNAs (13, 14). Although trypanosomes lack canonical DNA sequence-specific transcription factors, they do harbor histone modifications and the histone-interacting enzymes required to write, erase, and read specific histone modifications (15, 16). Recent work has demonstrated that there are a large number of histone-interacting proteins that form specific complexes in bloodstream forms, and that many of these complexes localize to sites of transcription initiation and termination (17). Perturbation of histone-interacting complexes in trypanosomes has been shown to affect immune evasion (18–23), cell cycle (24), and differentiation (23–25) processes.

We previously showed that small-molecule and genetic inhibition of chromatin-interacting bromodomain proteins in bloodstream parasites induces transcriptome reprogramming that shares similarities to transcriptomic changes that occur during the transition from the bloodstream to the procyclic form of the parasite (23) in the insect midgut. Trypanosome bromodomain proteins bind to acetylated lysines (26, 27) and Bdf1-6 (17, 23, 26) and have been shown to localize to transcription start sites. Another bromodomain protein (Bdf7) localizes to termination sites (17). In addition, occupancy of the bromodomain protein Bdf3 is dynamic, as parasites differentiate from bloodstream to procyclic forms, with occupancy peaking at 3 h postdifferentiation (25). The small-molecule bromodomain inhibitor I-BET151 binds to TbBdf2 and TbBdf3 and may bind additional bromodomain proteins, although this has not been formally tested (23). Treatment of bloodstream parasites with I-BET151 induces remodeling of the parasite surface wherein VSG proteins are downregulated and procyclin protein is expressed on the cell surface. In addition, large numbers of transcripts that are upregulated or downregulated during differentiation to the procyclic stage are similarly up- or downregulated in I-BET151-treated bloodstream parasites (23).

Accompanying the differentiation-like changes in I-BET151-treated parasites is a loss of monoallelic expression of VSG genes (23). Immune evasion of the mammalian antibody response depends on the expression of only one VSG protein on the parasite surface at a time, and the rest of the large repertoire of VSG genes are silenced. I-BET151 treatment increases expression of VSG genes from silenced bloodstream expression sites (BESs) and also increases the expression site-associated genes (ESAGs) from these same sites. Metacyclic VSG genes and VSG genes at other sites in the genome are also derepressed in I-BET151-treated bloodstream parasites.

While the effect of small-molecule bromodomain inhibition has been characterized in bloodstream parasites (23), the effect of I-BET151 treatment in insect-stage parasites is unknown. In order to further understand how bromodomain inhibition affects the transcriptome of insect-stage parasites, we performed transcriptome sequencing (RNA-seq) time courses on I-BET151-treated midgut-stage procyclic cells. Our analysis shows that large numbers of transcripts are altered in I-BET151-treated procyclic parasites and that drug-treated parasites share some features with parasites in the early metacyclic salivary gland stages. We also show that silencing of VSG genes is perturbed in I-BET151-treated parasites, demonstrating that bromodomain proteins are important for gene silencing in multiple stages of the parasite life cycle.

## RESULTS

### VSGs and ESAGs are among the most highly upregulated genes following bromodomain inhibition.

In order to investigate the effects of bromodomain inhibition in insect-stage parasites, we treated procyclic parasites with the previously validated bromodomain inhibitor I-BET151 (23). I-BET151 binds directly to Bdf2 and Bdf3 and may also bind other bromodomain proteins (23). Procyclic parasites were treated with I-BET151 over the course of 2 weeks, and samples were harvested for RNA-seq at 10 time points (0 h, 3 h, 6 h, 12 h, 24 h, 48 h, 3 days, 7 days, 10 days, and 14 days). Throughout the experiment, parasites were counted with a hemocytometer every 48 h and diluted to a concentration of 2 million parasites/mL in fresh medium every 2 days. I-BET151 or dimethyl sulfoxide (DMSO) was replenished at each passage. We observed that I-BET151-treated parasites consistently grew more slowly than DMSO-treated controls; however, the parasites continued to grow and remain viable throughout the experiment (see Fig. S1 in the supplemental material). The number of DMSO-treated parasites ranged from 1.5- to 3-fold higher throughout the experiment, with later time points showing more growth inhibition. Doubling times for DMSO-treated parasites ranged from 10.5 hours to 12.2 hours throughout the experiment, whereas doubling times for I-BET151-treated parasites ranged from 13.7 hours to 20.4 hours, with larger doubling times observed at 10 days or longer. No gross morphological abnormalities were observed during treatment.

**FIG 1:**
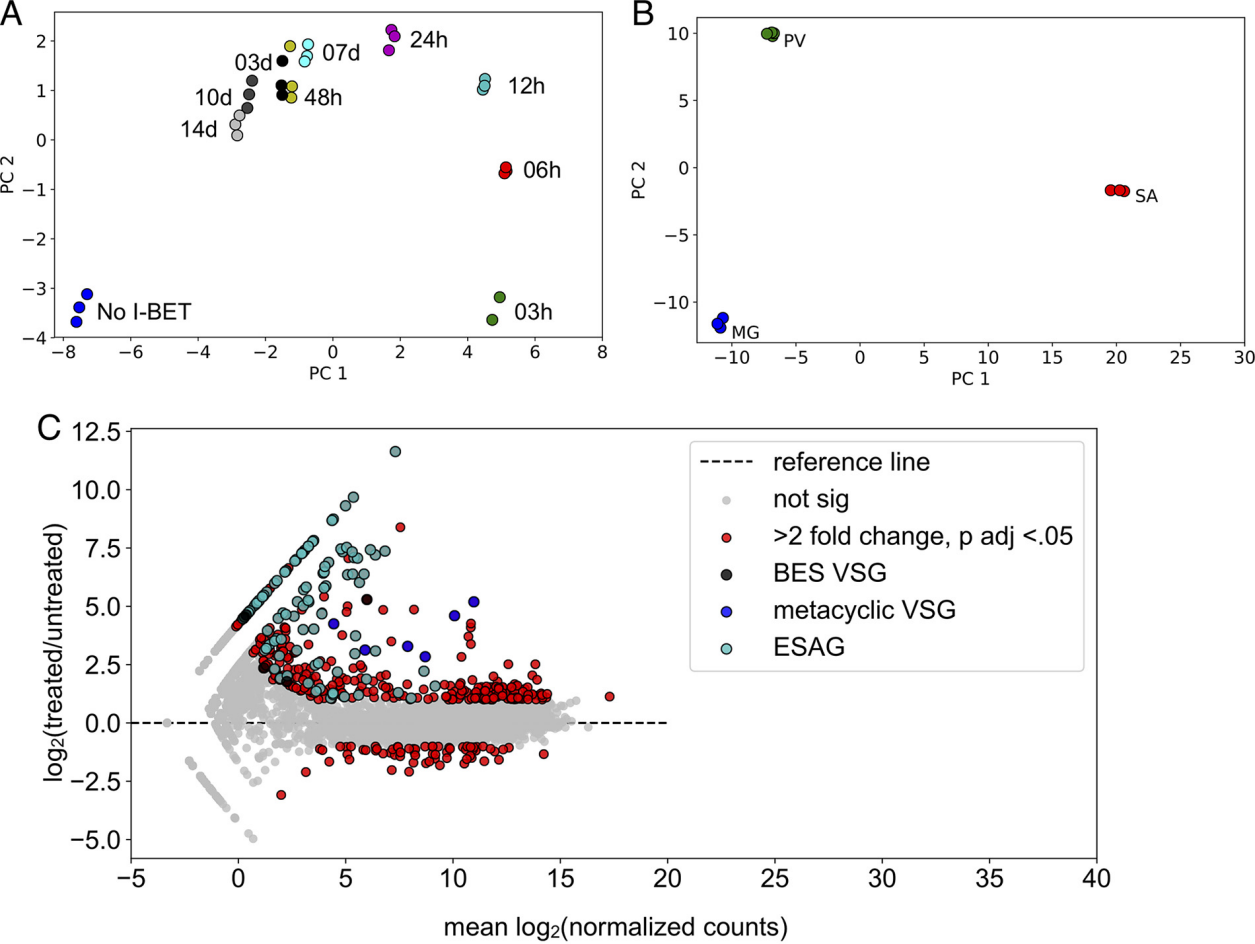
VSG genes and ESAGs are the among the most highly upregulated genes following I-BET151 treatment in insect-stage parasites. (A) PCA of RNA-seq samples for I-BET151-treated parasites at indicated time points. (B) PCA of RNA-seq samples from indicated tsetse fly organs using data from the work of Savage et al. (28). MG, midgut; PV, proventriculus; SA, salivary gland. (C) MA plot for parasites treated for 12 h with I-BET151 compared with untreated parasites. Red dots indicate differentially expressed genes, identified by DESeq, with a Benjamini-Hochberg-adjusted *P* value of <0.05 and a fold change of >2.

We first performed a principal-component analysis (PCA) to confirm that biological replicates clustered together (Fig. 1A). MA plots comparing DESeq-normalized transcript levels at 12 h of I-BET151 treatment with those of untreated parasites revealed a large number of genes with significantly altered transcript levels at this early time point (Fig. 1C). We observed that transcript levels for VSG genes and ESAGs were among the most highly upregulated in the data set. This is consistent with previous work in bloodstream parasites showing that I-BET151 treatment and RNA interference (RNAi)-mediated silencing of individual bromodomains causes an increase in transcript levels of silent VSG genes (23).

**FIG 2:**
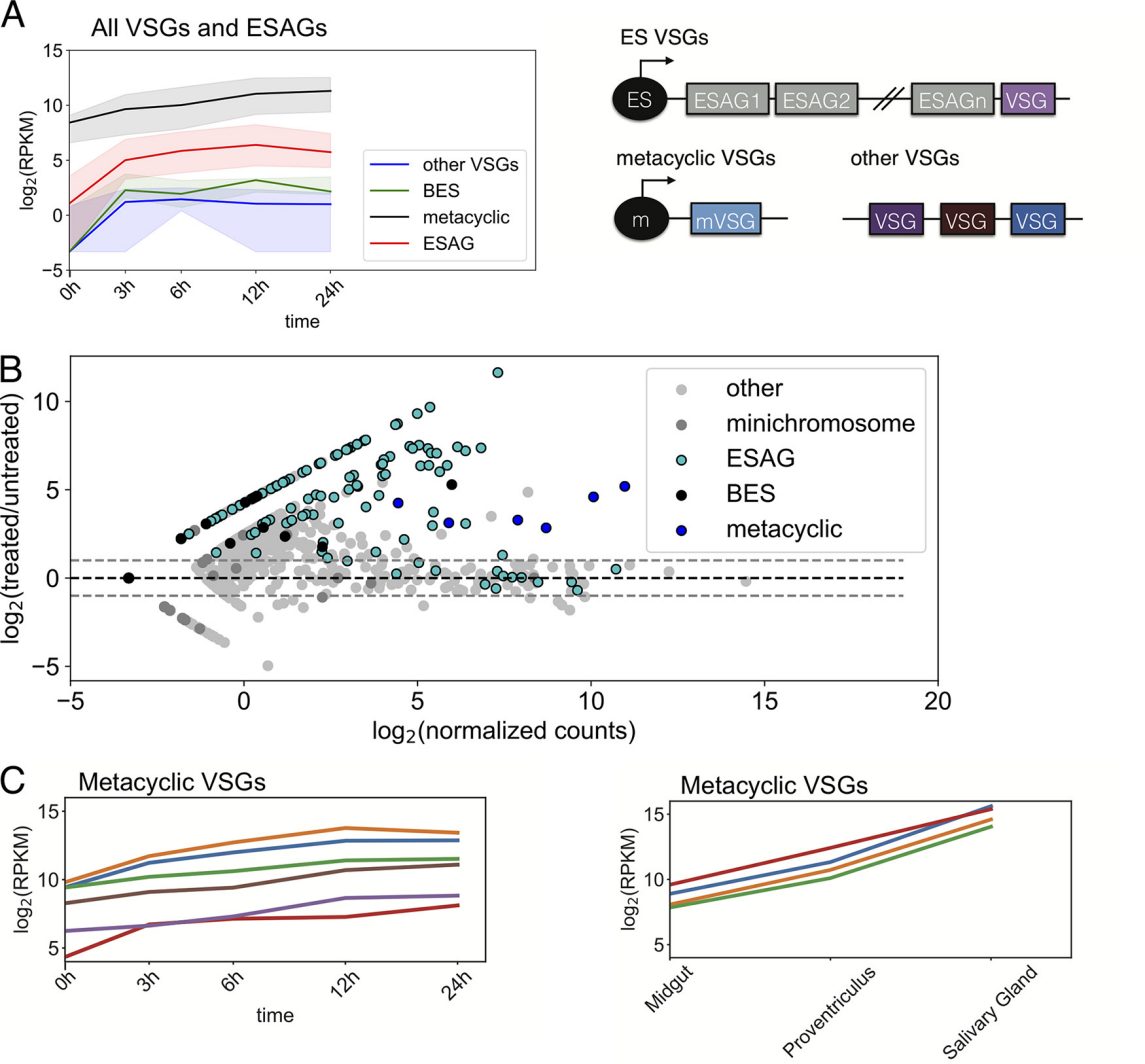
I-BET151 treatment in insect-stage parasites increases transcript levels of VSG genes and ESAGs that are silenced in wild-type parasites. (A) (Left) Median transcript levels for each indicated gene subset over 24 h of I-BET151 treatment. Shading represents the inner quartile range at each time point. (Right) Schematic of VSG gene locations in the genome. (B) MA plot for VSG gene subsets and ESAGs in parasites treated for 12 h with I-BET151 compared with untreated parasites. Dashed lines demarcate 2-fold differences in treated parasites compared to untreated parasites. (C) (Left) Transcript levels for 6 individual VSG genes with metacyclic promoters over 24 h of I-BET151 treatment. (Right) Transcript levels for 4 VSG genes in samples taken from the indicated organs of tsetse flies using data from the work of Savage et al. (28).

To further investigate the effect of I-BET151 treatment on VSG gene and ESAG transcript levels, we separated the VSG genes into subsets: those encoding VSGs within BESs, metacyclic VSG genes that contain specific promoters active in the salivary gland stage, and other VSG genes that are located elsewhere in the genome. We plotted the median expression level for each of these sets of genes and observed an increase in transcript levels for all of the VSG gene subsets and for ESAGs at the 12-h time point compared to untreated parasites (Fig. 2A and B). This is the phenotype observed in bloodstream parasites following bromodomain inhibition (23). We conclude from these data that bromodomain proteins are important for maintaining silencing of VSG gene transcripts in insect-stage procyclic parasites in addition to their established role in maintaining VSG gene silencing in bloodstream-stage parasites.

### Metacyclic VSG gene transcript levels are increased following I-BET151 inhibition.

Bromodomain inhibition in bloodstream parasites results in changes in the transcriptome that resemble many of the changes in transcript levels that occur as bloodstream parasites transition to the procyclic stage (23). We wanted to test the model that bromodomain inhibition might initiate a default program that results in transcript changes consistent with developmental progression. This model predicts that inhibition of bromodomain proteins in gut-stage procyclic parasites might cause transcriptome changes similar to those that occur as gut-stage parasites migrate to the proventriculus and from there to the salivary gland. To investigate this question, we took advantage of an existing RNA-seq data set generated from parasites living in the fly gut, proventriculus, and salivary gland (28), which we refer to here as the insect-stage-development data set. We obtained fastq files from the insect-stage-development data set and analyzed them using our RNA-seq pipeline to ensure the data set was comparable to the bromodomain inhibition data set that we generated. A PCA demonstrated that the replicates from the insect-stage-development data set clustered together following analysis with our pipeline (Fig. 1B). One of the most prominent changes in gene expression following transition to the salivary gland is the upregulation of VSG genes with metacyclic promoters that facilitates transition to a bloodstream environment (29). We plotted the expression of VSG genes with metacyclic promoters following I-BET151 treatment and compared it to the expression of metacyclic VSG genes in the insect-stage-development data set (Fig. 2C). As expected, we observed that the expression of metacyclic VSG genes increased sharply as parasites transitioned to the salivary gland. We also observed an increase in transcript levels of metacyclic VSG genes following I-BET151 treatment (note that the metacyclic VSG genes are named differently in the two different strains used) (Fig. 2C). These data indicate that I-BET151 treatment and developmental progression have similar effects on transcript levels for the metacyclic VSG gene subset. However, since most VSG gene subsets show increased transcript levels following I-BET151 treatment, we also investigated the behavior of other sets of genes known to be associated with developmental progression.

### Some gene sets associated with developmental progression show changes in transcript levels following I-BET151 treatment.

**FIG 3:**
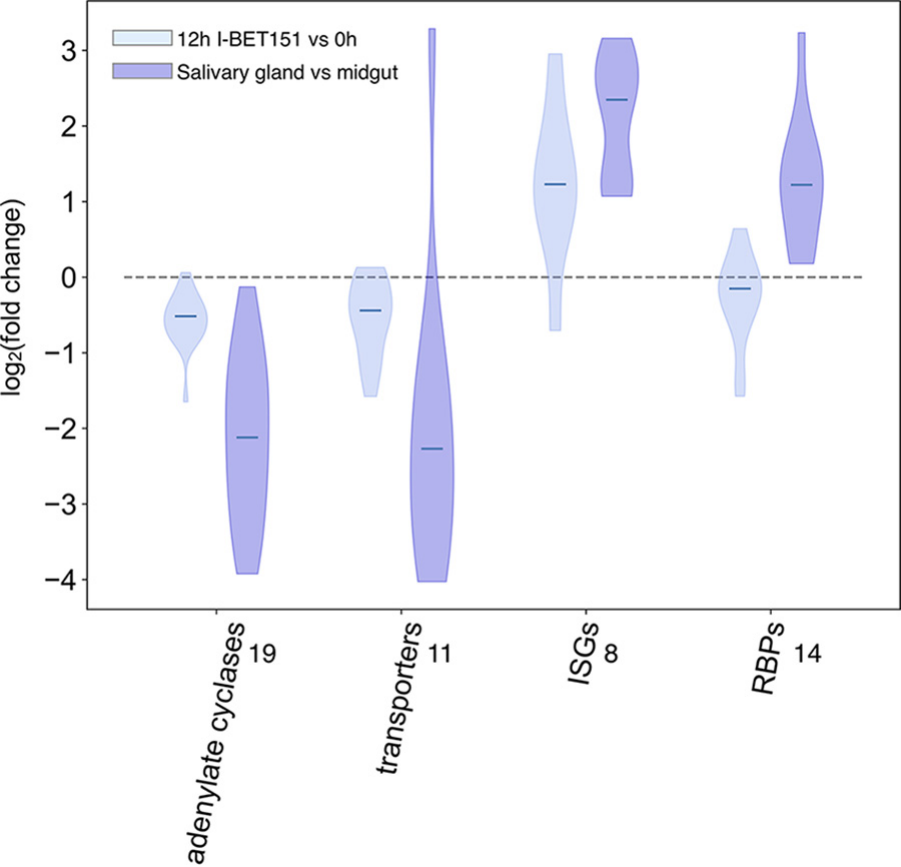
Transcript levels for some gene sets associated with insect-stage developmental progression are also altered following I-BET151 treatment. Violin plot for transcript levels of adenylate cyclases and transporters that are downregulated during insect-stage developmental progression and transcript levels of ISG and RBP genes that are upregulated during developmental progression. Data are plotted as log_2_(I-BET151 [12 h]/untreated [0 h]) and log_2_(salivary gland/midgut) using data on parasites harvested from the midgut and salivary gland from the work of Savage et al. (28). Data for all gene sets shown were statistically significant using GSEA (Table S1). Numbers to the right of the *x* axis labels indicate the numbers of genes in the gene set analyzed.

We next investigated whether other gene sets identified in the insect-stage-development data set were also altered following treatment with I-BET151. Savage et al. (28) identified a number of gene sets with altered transcript levels as parasites progressed from the midgut to the salivary gland. These included a set of adenylate cyclases, transporters, RNA binding proteins, and surface proteins (28). We separated each gene set into those that increased during developmental progression and those that decreased. We performed gene set enrichment analysis (GSEA) to see if any of these gene sets were overrepresented in the set of differentially expressed genes following treatment with I-BET151. Genes with transcript levels that change early in the time course are likely a more direct result of bromodomain inhibition than changes that occur later, so we focused on genes that were differentially expressed by 12 h following bromodomain inhibition. Of the gene sets tested this way, the set encoding adenylate cyclases and transporters that were downregulated during developmental progression also showed downregulation following bromodomain inhibition (Fig. 3) and showed significant enrichment in the GSEA (Table S1).

In addition, many of the invariant surface glycoproteins (ISGs) that were identified as upregulated in the insect-stage-development data set were also upregulated following I-BET151 treatment (Fig. 3). Although upregulated RBPs from the data set reported by Savage et al. (28) were significantly enriched, as determined by GSEA in I-BET151-treated parasites, some members of this set increased while others decreased, and thus, no clear-cut pattern for expression emerged for this group (Fig. 3). Thus, bromodomain inhibition in procyclic parasites results in some transcript level changes that are similar to those that occur during insect-stage developmental progression. However, we note that patterns of gene expression that are considered hallmarks of the transition from parasites living in the gut to those in the salivary glands do not show significantly altered levels in I-BET151-treated procyclic parasites. Specifically, the EP genes that are downregulated and the Brucei alanine rich protein (BARP) genes that are upregulated during the transition to the epimastigote stage do not show significantly altered transcript levels following I-BET151 treatment. We verified that procyclin expression was not altered in procyclic parasites following I-BET151 treatment using a previously validated *EP1/GFP* reporter parasite line (30) (Fig. S2). We observed no difference in *EP1/GFP* expression in I-BET151-treated parasites (Fig. S3).

### Parasites treated with I-BET151 share transcriptomic features with early metacyclic parasites.

**FIG 4:**
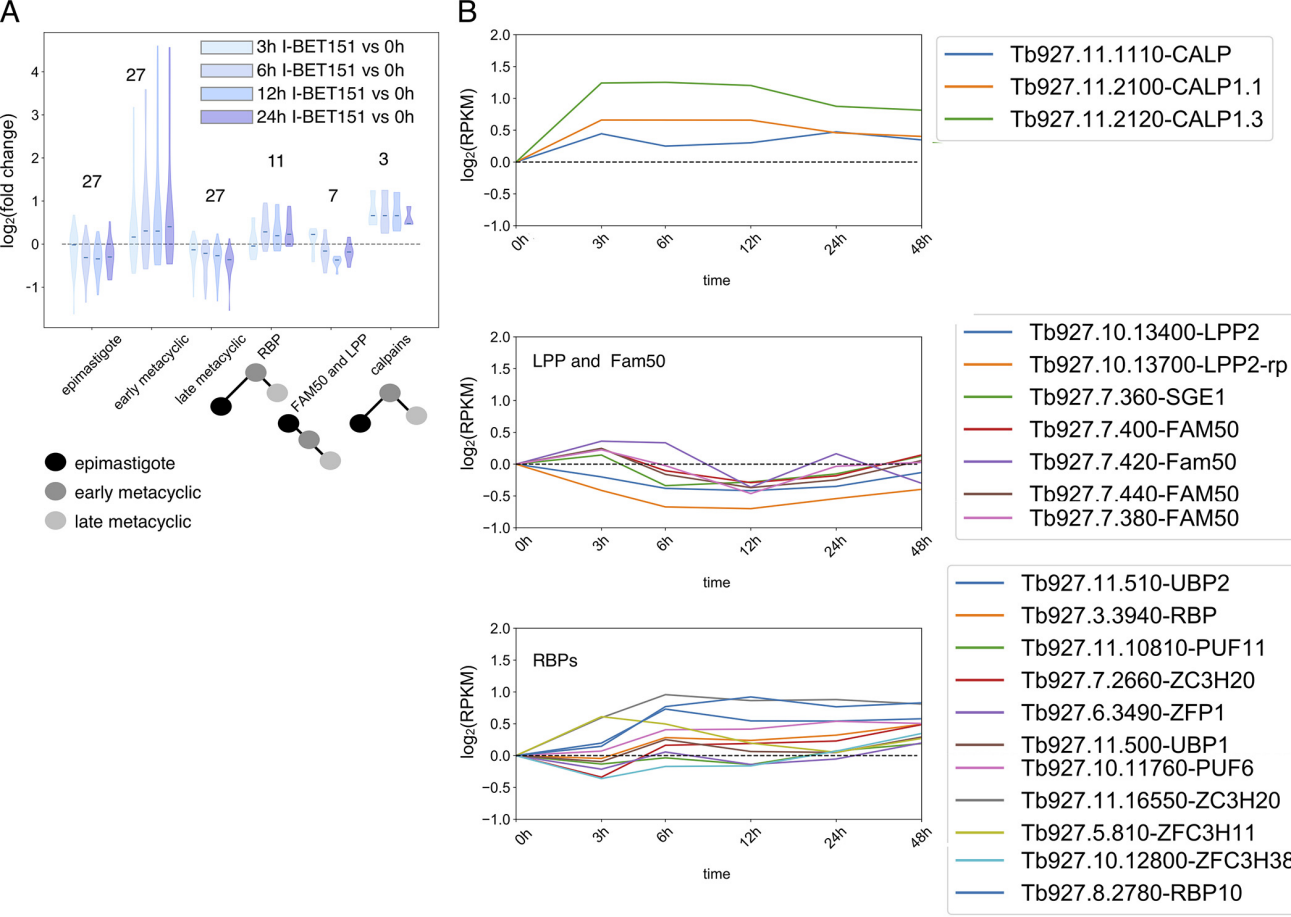
I-BET151-treated parasites share some transcriptomic features of early metacyclic parasites. (A) Violin plot showing transcript levels of indicated gene sets defined by Vigneron et al. (31) in parasites over 24 h of I-BET151 treatment. Numbers over gene sets indicate the numbers of genes in set. Data are plotted as log_2_(I-BET151-treated/untreated [0 h]) using normalized counts. Blue shading indicates time of I-BET151 treatment. Schematics under the labels represent transcript levels for each group during development as measured by Vigneron et al. (31), where a black node indicates transcript level at the epimastigote stage, a dark gray node represents transcript level in early metacyclic parasites, and a light gray node indicates transcript level in late metacyclic parasites. (B) Normalized transcript levels for the indicated genes following I-BET151 treatment. Each gene set was identified by Vigneron et al. (31) as having altered expression specifically in early-metacyclic-stage parasites.

We next compared I-BET151-induced transcriptomic differences with those described for a single-cell sequencing data set cataloguing developmental progression in insect-stage parasites (31). The advantage of this data set is that the metacyclic stages are separated into more subsets than in the study by Savage et al. (28). Vigneron et al. (31) separated parasites into 3 broad categories, epimastigote, meta 1, and meta 2, with meta 1 representing early metacyclic parasites and meta 2 representing later-stage metacyclic parasites. Each broad category was selected for a set of biomarker genes that represented highly differentially expressed genes. We performed GSEA for these 3 developmental-group biomarker genes on our I-BET151 data set. While all gene groups were significantly enriched in our I-BET151 differentially expressed data set, the meta 1 biomarker gene set was specifically upregulated (Fig. 4A; Fig. S4). Vigneron et al. (31) also catalogued the expression of a set of signature genes during developmental progression. These include a set of genes for RNA-binding proteins (RBPs), the Fam50 gene, lipid phosphate phosphatase (LPP) genes, Fam10 genes, and calpain genes. With the exception of the calpains, all these gene sets were significantly enriched in our I-BET151-treated parasites by GSEA (Table S1). Although the calpain genes did not meet our false discovery rate (FDR) threshold for GSEA, visual inspection of this gene set showed an increase in transcript levels for all 3 genes, which peak in expression at the early meta 1 stage in developing parasites. Vigneron et al. (31) found that a set of RBP genes peak at the meta 1 stage, while the expression of the Fam50 and LPP gene set decrease at the meta 1 stage. I-BET151-treated parasites also showed increases in these RBP genes, while the Fam50 and LPP gene sets briefly increased and then decreased by 12 h (Fig. 4A and B). While Vigneron et al. (31) observed an increase in transcript levels for Fam10 genes in late meta1 and early meta 2 stages, we did not see an upregulation of that gene set in I-BET151-treated parasites.

Overall, we conclude that a model wherein bromodomain inhibition leads to a default program of developmental progression is too simplistic to describe the observed data. Our analysis shows that I-BET151-treated parasites share transcriptomic features with early metacyclic parasites, including upregulation of the meta 1 biomarker gene set (Fig. 4A) and changes in gene expression for adenylate cyclases, transporters, RBPs, calpains, LPP, and Fam50 genes. However, some hallmarks of developmental progression are not observed, including upregulation of *BARP*, downregulation of the *EP* genes, and upregulation of the Fam10 gene set, which has been shown to occur in the transition from early to late metacyclic stages (31).

### Clustering analysis reveals that differentially expressed genes in I-BET151-treated parasites can be grouped into 6 distinct expression patterns.

**FIG 5:**
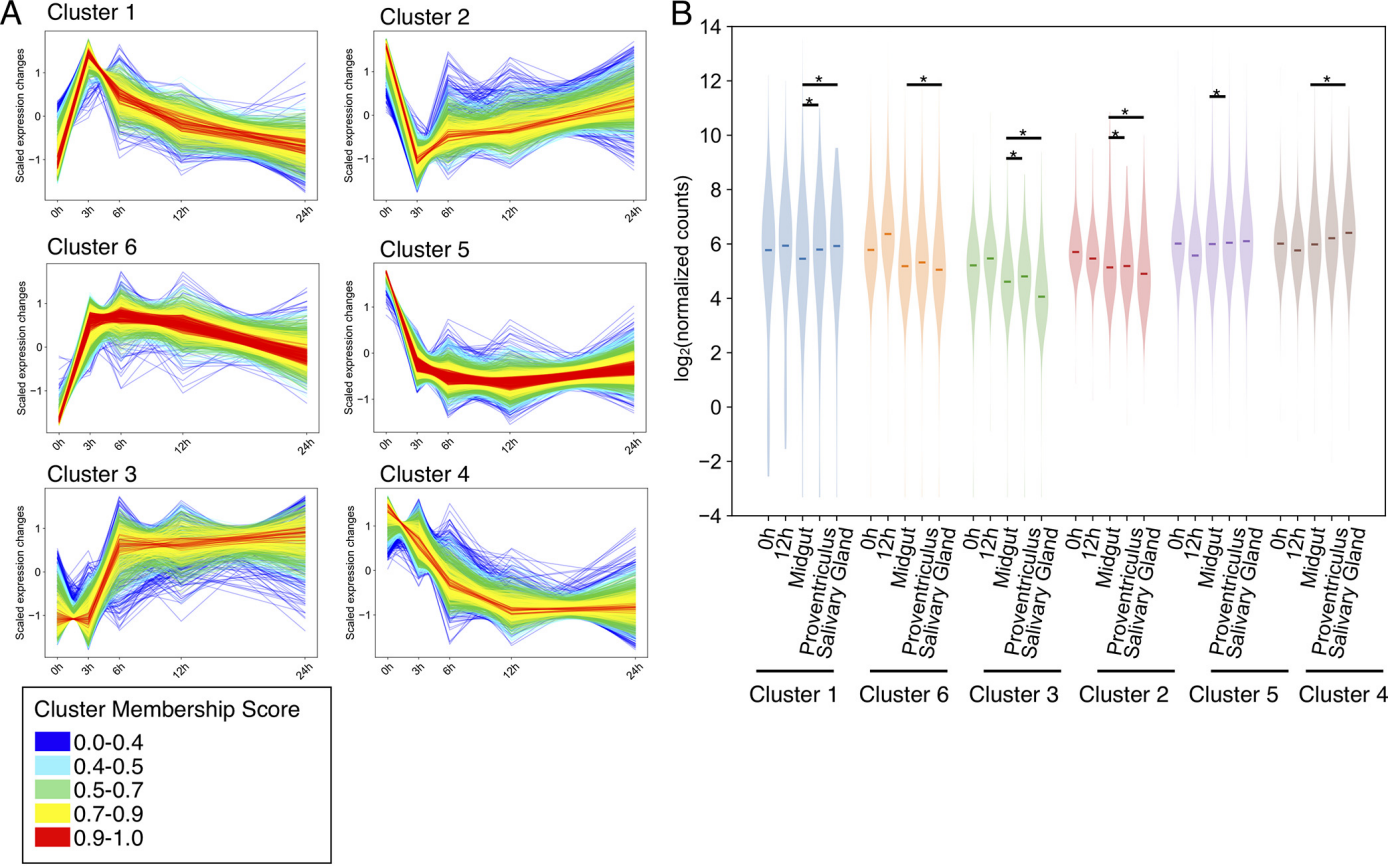
Differentially expressed genes in I-BET151-treated parasites show distinct patterns of expression. (A) DESeq normalized transcript levels of differentially expressed genes following I-BET151 treatment, clustered based on expression pattern and timing. Transcript levels are scaled such that the mean is 0 and the standard deviation is 1. (B) Violin plot comparing changes in transcript levels for each cluster in panel A following 12 h of I-BET151 treatment or during insect-stage developmental progression using data for midgut, proventriculus, and salivary gland parasites taken from the work of Savage et al. (28). Stars indicate clusters identified as enriched by GSEA with an FDR of <0.1 in the insect-stage-developmental-progression data set.

We next analyzed the I-BET151 data set to identify groups of genes whose transcript levels change with similar timing and magnitude. As we were particularly interested in early effects following bromodomain inhibition, we identified all differentially expressed genes across 5 time points in the first 24 h following I-BET151 treatment (0 h, 3 h, 6 h, 12 h, and 24 h) using a likelihood ratio test (LRT). This analysis revealed that a large number of 6,548 genes were differentially expressed in the first 24 h after bromodomain inhibition with a Benjamini-Hochberg-adjusted *P* value of <0.01. We then used the Mfuzz program (32) to group each of these genes into one of six clusters of genes (Fig. 5). We identified three clusters of upregulated genes (clusters 1, 3, and 6) and three clusters of downregulated genes (clusters 2, 4, and 5) (Fig. 5). Clusters 1 and 2 have a sharp change in expression at 3 h that rapidly reverses toward the starting transcript levels. Clusters 5 and 6 have sharp changes in expression at 3 h, and this change persists throughout the first 24 h of I-BET151 treatment. Finally, clusters 3 and 4 have more gradual changes in expression levels that also persist for the first 24 h. Of all the upregulated VSG genes that were observed, the majority fell within cluster 3, which shows a more gradual increase in gene expression than clusters 1 and 6. We conclude that bromodomain inhibition in insect-stage parasites results in large changes in gene expression, many of which occur quite early, within 6 h of treatment.

We next looked at whether the clusters of genes we identified following I-BET151 treatment were enriched in the differentially expressed genes from the insect-stage-development data set (28). To do this, we first filtered each cluster for a membership correlation value of 0.7, which resulted in 2,549 genes spread out among the 6 clusters (Table S7). We then interrogated the insect-stage-development data set to see if these high-membership clustered gene sets were overrepresented in parasites developing in the proventriculus or the salivary gland. All the high-membership clusters were significantly overrepresented by GSEA in parasites harvested from either the proventriculus or the salivary gland compared to midgut parasites (Table S1). However, only 4 clusters showed changes in I-BET151-treated parasites consistent with the direction of change during developmental progression. Specifically, clusters 1, 3, and 6 showed median expression values that increase with I-BET151 treatment; an increase in median expression values for these clusters was also observed as parasites progressed from the midgut to the proventriculus. Cluster 1 showed median expression values that increase with I-BET151 treatment; an increase in median expression value was also observed for this cluster as parasites progressed from the midgut to the salivary gland. Finally, a decrease in median expression value was observed for genes in cluster 2 following I-BET151 treatment, and a decrease in expression value was also seen for this cluster as parasites progressed from the midgut to the salivary gland.

**FIG 6:**
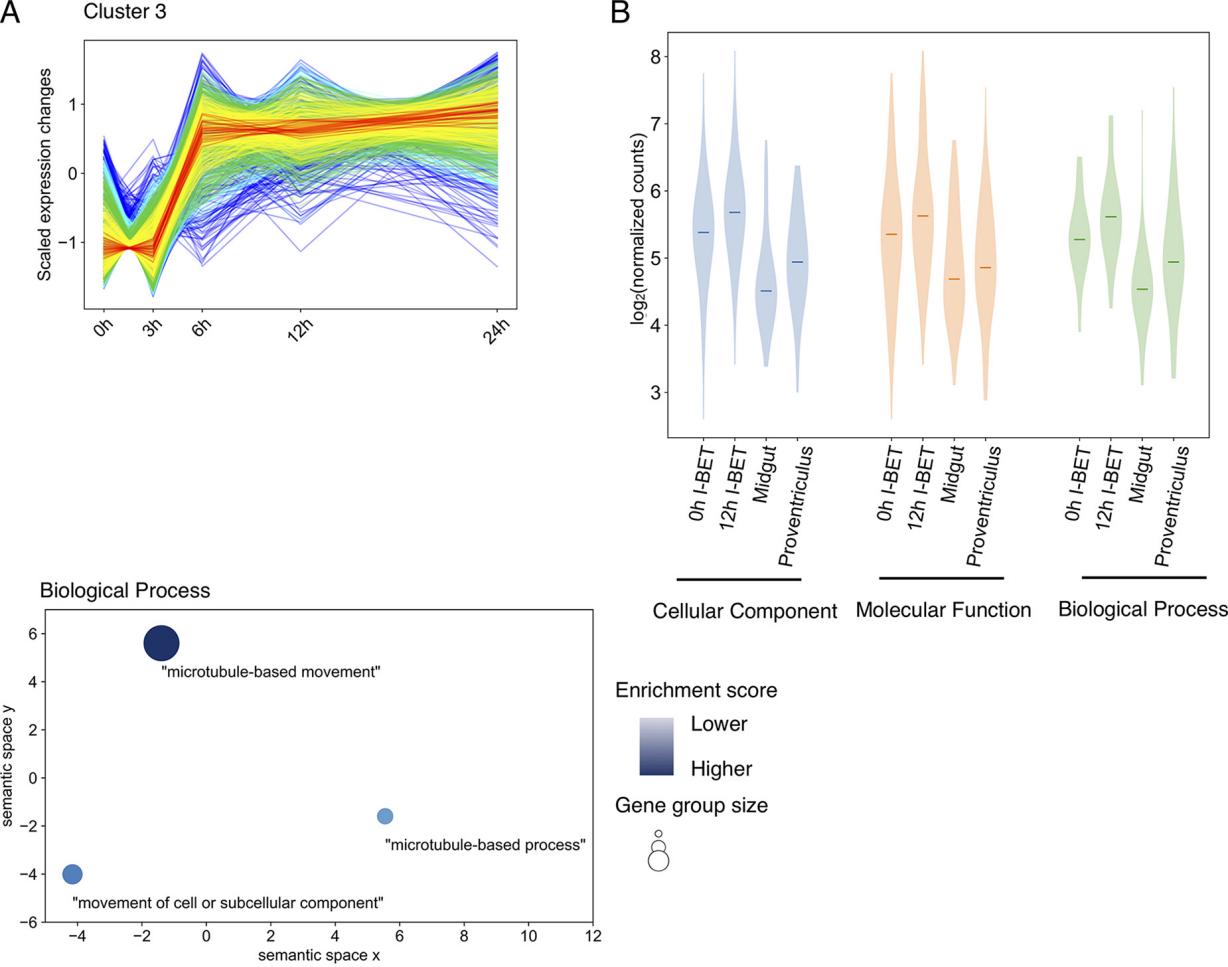
Genes involved in movement and microtubule-based processes are upregulated in I-BET151 parasites and in parasites transitioning from the midgut to the proventriculus. (A) (Top) Same as in Fig. 5A. Bottom, REVIGO (80) plot of GO enrichment analysis performed on cluster 3. (B) Violin plot for genes identified as enriched by GO analysis of cluster 3. Transcript levels for I-BET151-treated parasites are compared with transcript levels in midgut and proventriculus stage parasites using data from the work of Savage et al. (28).

We performed a gene ontology (GO) analysis on each of the high-membership clusters we identified in the I-BET151 treatment data set. We filtered the GO terms using a Benjamini-Hochberg-adjusted *P* value of less than 0.05 and an enrichment score greater than 3 (Table S5). While this analysis was not revealing for many of the clusters, cluster 3 was particularly enriched for GO terms having to do with cell movement, particularly with microtubule-based processes (Fig. 6 and Fig. S5). This is consistent with a meta 1 developmental phenotype as defined by Vigneron et al., who found that genes associated with cell movement were particularly upregulated at this developmental stage (31). Cluster 3 was more slowly upregulated following bromodomain inhibition, and the upregulation persisted for the first 24 h of treatment (Fig. 6A). We plotted the expression levels for genes within each GO-enriched group for cluster 3 following bromodomain inhibition and during insect-stage developmental progression (Fig. 6B). These results demonstrate that the median expression level for genes associated with movement in cluster 3 increases following I-BET151 treatment and as parasites transition from the midgut to the proventriculus. We conclude that bromodomain inhibition causes changes in transcript levels for genes associated with microtubule-related processes important for cell movement, which are also processes involved in developmental progression.

### Gene ontology analysis reveals that I-BET151 treatment causes changes in transcript levels for genes associated with a wide array of cellular processes.

**FIG 7:**
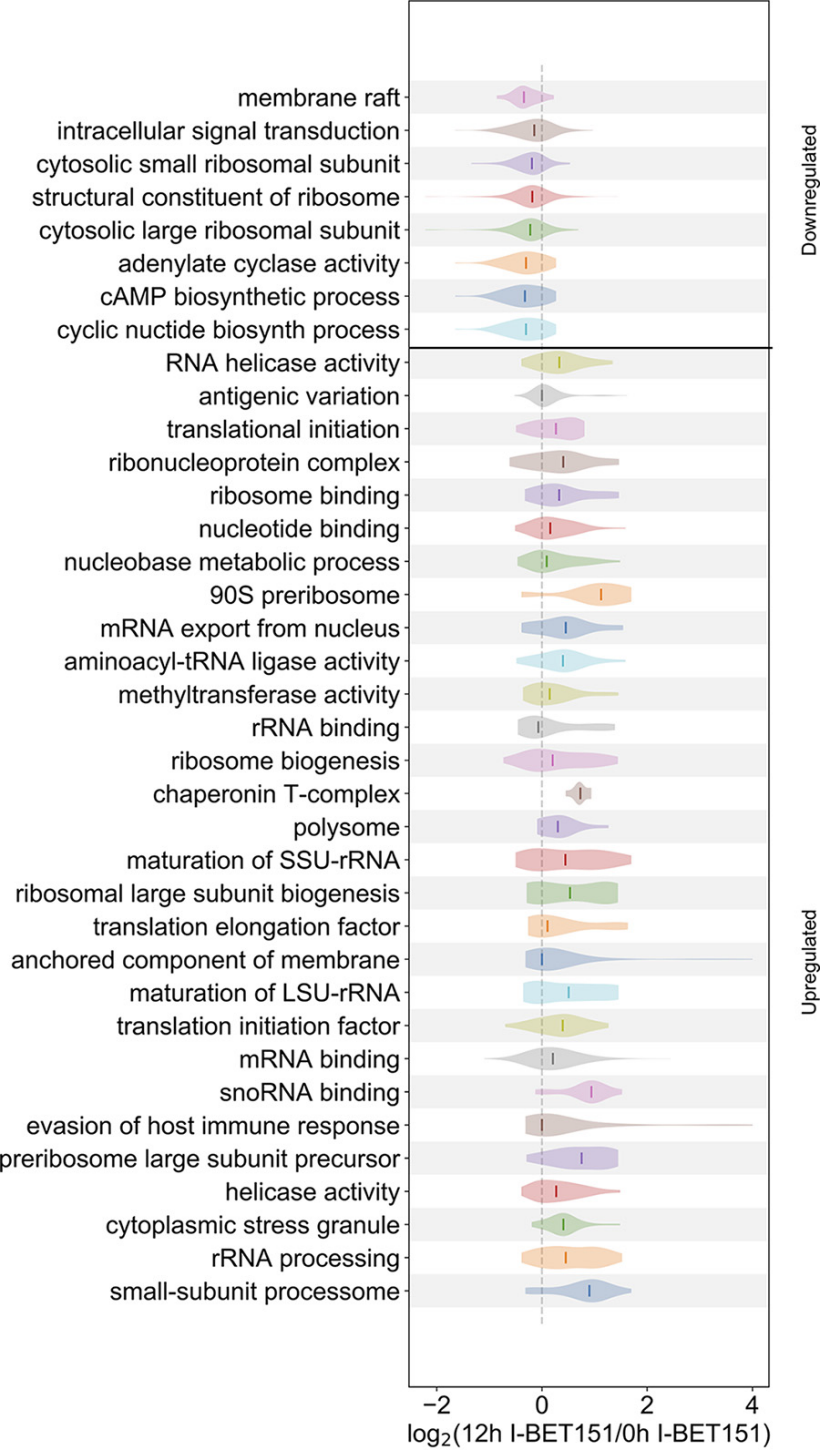
Diverse biological processes affected by I-BET151 treatment in insect-stage parasites. Violin plot showing transcript levels of parasites treated for 12 h with I-BET151 compared with untreated parasites (0 h) expressed as log_2_(12-h I-BET151/0-h IBET151) using normalized counts for GO sets with an FDR of <0.1 by GSEA.

To better capture the full array of transcriptome changes that occur following I-BET151 treatment in insect-stage parasites, we performed GSEA on sets of genes grouped by GO. In this analysis, we once again focused on genes that were differentially expressed between the 0-h time point and 12 h of I-BET151 treatment. This analysis revealed that a large number of gene sets were affected. Biological processes associated with antigenic variation and evasion of the host immune response, adenylate cyclase activity, translational initiation, rRNA processing, intracellular signal transduction, translation initiation factor activity, cyclic AMP (cAMP) biosynthesis, metabolic processes involving nucleobase containing compounds, and cyclic nucleotide biosynthetic processes were all significantly enriched in the differentially expressed gene set following 12 h of bromodomain inhibition (Table S1 and Fig. 7). The large number of gene sets affected by I-BET151 treatment indicates that bromodomain inhibition has effects on a diverse set of biological processes and that the transcriptomic differences observed following treatment likely reflect direct and indirect effects of the drug, even at early time points.

### I-BET151 driven transcriptome changes vary for different life cycle stages.

**FIG 8:**
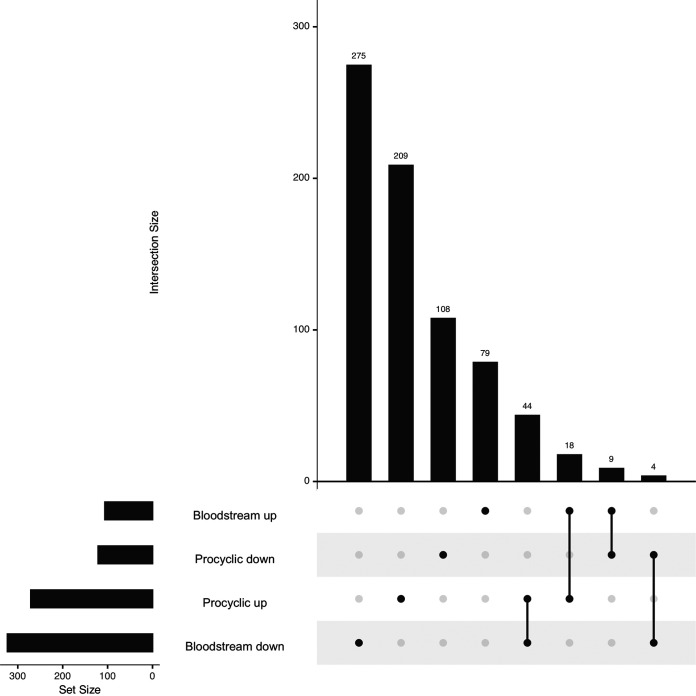
Many transcriptomic changes initiated by I-BET151 treatment are life cycle stage specific. UpSet plot showing differentially expressed genes identified by DESeq with a Benjamini-Hochberg-adjusted *P* value of <0.05 and a fold change of >2 up or down in bloodstream and procyclic-stage parasites. Data from bloodstream parasites were generated from the work of Schulz et al. (23).

To try to understand whether the effects of bromodomain inhibition are context dependent, we compared the most highly upregulated and downregulated genes following bromodomain inhibition in bloodstream-form parasites and procyclic-form parasites. To do this, we took advantage of a previously published RNA-seq time course on bloodstream-form parasites treated with I-BET151 (23). We used DESeq2 to ascertain which differentially expressed genes had alterations in transcript levels of >2-fold (up or down) at any time point compared to untreated parasites. We separated transcripts into those that were increased and decreased following bromodomain inhibition and generated an UpSet plot to ascertain which genes were common to the bloodstream-stage data set and the procyclic-stage data set (Fig. 8). This analysis revealed that a large number of transcript levels are upregulated or downregulated only in one of the life cycle stages analyzed. Of the 746 transcripts that showed large changes following bromodomain inhibition, only 75 were affected by I-BET151 treatment in both procyclic and bloodstream forms. Of these, the largest group were 44 genes whose transcript levels are downregulated in bloodstream forms and upregulated in procyclic forms following I-BET151 treatment (Table S6). This group includes 6 ISGs, which are downregulated during differentiation from bloodstream to procyclic forms (5) and upregulated in salivary gland parasites (28).

The next largest group of 18 genes with large changes in both bloodstream and procyclic bromodomain-inhibited parasites were upregulated in both forms. This group included 4 procyclin associated genes (PAGs), 1 PAD (protein associated with differentiation) gene (*PAD3*), and a number of other genes that are highly upregulated in parasites transitioning from the bloodstream form to the procyclic form (Tb927.8.520, Tb927.8.480, Tb927.3.590, Tb927.10.9550, Tb927.11.4700, Tb927.2.3460, Tb927.8.1270, Tb927.3.2750, and Tb927.10.2310) (5). In this group, an adenosine transporter gene (Tb927.3.590), a prostaglandin F synthase gene (Tb927.11.4700), and a cysteine peptidase family gene (Tb927.2.3460) were highly upregulated in parasites moving from the midgut to the salivary gland. Nine genes were upregulated in bromodomain-inhibited bloodstream forms and downregulated in procyclic forms, while four genes were downregulated in bloodstream forms and upregulated in procyclic forms. We conclude that the effect of I-BET151 on the transcriptome is highly context dependent based on life cycle stage. With the exception of the VSG genes and ESAGs, the sets of genes that are upregulated or downregulated following I-BET151 treatment in procyclic versus bloodstream forms do not contain large overlaps (Fig. 8).

### Prolonged I-BET151 treatment results in partial transcriptional adaptation.

We observed that prolonged treatment with I-BET151 resulted in parasites that clustered closer to untreated parasites in our RNA-seq PCA plot (Fig. 1A). To investigate the idea that parasites transcriptionally adapt to I-BET151 treatment, we plotted the expression of 10 individual genes that were the most highly upregulated or downregulated at the 12-h treatment time point with an adjusted *P* value of <0.05 (Fig. S6A). We found that the majority of the genes that were upregulated at 12 h following treatment showed a decrease in transcript levels at later time points. Similarly, the majority of genes that were downregulated at 12 h showed increases in transcript levels at later time points. However, in neither case did the transcript levels return to their starting levels for most of the genes examined (starting levels are represented by the dashed lines in Fig. S6A). To show this in a more general way, we plotted the median expression level for the top 20% of genes that were upregulated at the 12 h time point and the top 20% of genes that were downregulated at the same time point (Fig. S6B). These data show a similar trend, with transcript levels rebounding but remaining slightly higher or lower than their starting state (Fig. S6B). To examine adaptation for all differentially expressed genes, we divided our differentially expressed genes into different bins using their fold changes at 12 h of I-BET151 treatment compared to 0 h. For all the gene groups examined, we observed a reversion of transcript levels that never returned to the starting state (represented by the dashed lines in Fig. S6C). We conclude that parasites transcriptionally adapt to I-BET151 and revert to a more “procyclic” starting state but that this reversion is incomplete, and parasites retain altered transcript levels for the majority of differentially expressed genes during prolonged I-BET151 treatment.

## DISCUSSION

Bromodomain inhibitors have garnered quite a bit of attention in recent years because of their potential as anticancer therapeutics. BET (bromodomain and extraterminal) inhibitors have been shown to inhibit tumor growth in a number of models, including acute myeloid leukemia (AML) (33–37), prostate cancer (38), neuroblastoma (39), and breast cancer (40). In parasites, bromodomain inhibitors have been shown to bind to bromodomain proteins in T. brucei, Trypanosoma cruzi, Plasmodium falciparum, and Leishmania donovani (23, 27, 41–44). Inhibition of bromodomain proteins in parasites has been shown to affect differentiation processes in multiple parasite systems (23, 45, 46), and therapeutic strategies targeting chromatin interacting proteins have also been proposed and/or demonstrated for trypanosomiasis (23, 30), Chagas disease (47–49), schistosomiasis (50, 51), toxoplasmosis (52, 53), leishmaniasis (54), and malaria (55, 56).

While the biological effects of the bromodomain inhibitor I-BET151 have been documented for bloodstream stage T. brucei parasites (23), nothing is known about the effect of bromodomain inhibition by this molecule at other parasite life cycle stages. While changes in transcript levels are observed for a large number of genes in I-BET151-treated insect-stage parasites, these changes do not result in complete growth arrest, nor do they kill the cell. Instead, I-BET151-treated parasites grow more slowly than DMSO-treated parasites. This might be the result of changes in transcript levels of metabolic genes (Fig. 7), but this could only be confirmed with further metabolomic studies. While I-BET151-treated parasites remain viable in culture, it is hard to know how the observed growth defects would translate to growth within the fly midgut. Since few parasites manage the passage to the salivary gland, a subtle growth defect might be sufficient for the fly to clear the infection. On the other hand, since the effects of I-BET151 in bloodstream forms is reversible (23), parasites might revert to normal growth if the I-BET151 pressure was removed following transfer to the fly.

The work presented here indicates that perturbations in VSG gene silencing following treatment with I-BET151 occurs in both bloodstream and insect-stage parasites (Fig. 2), whereas the effects on polymerase II (PolII)-driven genes are more context dependent and depend on the life cycle stage (Fig. 8). I-BET151 inhibition in bloodstream parasites causes remodeling of cell surface proteins, wherein I-BET151-treated parasites show a decrease in VSG protein on the surface and a concomitant increase in procyclin protein (23). While metacyclic VSG genes were upregulated in I-BET151-treated insect-stage parasites, we detected no differences in the expression of procyclin (Fig. S3), indicating that extensive remodeling of the cell surface is unlikely to occur following bromodomain inhibition. SAP1 of T. brucei (TbSAP1) was recently identified as an important repressor of metacyclic VSG expression (22). We observed reasonably high and unvarying transcript levels of TbSAP1 following bromodomain inhibition, and it is possible that the presence of this protein prevented sufficient expression of metacyclic VSG genes to support surface remodeling. Alternatively, additional checkpoints might be required to remodel the cell surface that were not induced by treatment with I-BET151. In bloodstream parasites, loss of VSG gene silencing has been tied to life cycle progression (57, 58). In parasites undergoing metacyclogenesis, all metacyclic VSG genes are upregulated prior to commitment to one particular metacyclic VSG (59). However, because the VSGs have not been as extensively catalogued in the parasite strain used for the insect developmental progression data set, it is unclear whether loss of VSG silencing for nonmetacyclic VSG genes is observed during insect-stage developmental progression.

I-BET151-treated procyclic parasites share some features with insect stage-differentiating parasites. I-BET151-treated parasites downregulate a set of adenylate cyclases that are also downregulated in salivary gland parasites (Fig. 3). These include flagellar receptor adenylate cyclases *ACP3-5* (*Tb927.10.13040*, *Tb927.10.13740*, and *Tb927.7.7470*) and a large number of gene related to expression site associated genes (GRESAGs). ACP3-5 are procyclic-stage-specific flagellar proteins (60), and other members of this family (ACP1, -2, and -6) have been shown to be important for social motility (61). ACP2 (Tb927.10.16190) and ACP6 (Tb927.9.15660) were also differentially expressed in our I-BET151-treated parasites. Salivary gland parasites also upregulate genes for invariant surface glycoproteins (ISGs) (62, 63) in preparation for transition to the bloodstream stage, and we found that this set of genes was also upregulated in I-BET151-treated parasites. The expression of ISG genes has recently been shown to be important for surviving the complement defense system in the mammalian bloodstream (64). Several glucose transporters important for the glycolytic pathway are also downregulated as parasites differentiate within the insect, and we found that the THT1E (Tb927.10.8450) and THT1 glucose transporters (Tb927.10.8440 and Tb927.10.8470) were downregulated in I-BET151-treated parasites. Other transporters, such as the nucleobase transporter Tb927.11.3620, cation transporter Tb927.11.8990, and pteridine transporter Tb927.10.9080, are downregulated in differentiating and I-BET151-treated parasites. This may reflect the different metabolic requirements of parasites at different stages within the fly compartment.

The elegant single-cell studies of Vigneron et al. (31) delineated key biomarkers for parasite development within the fly. Of these classified biomarkers, we found that the early metacyclic meta 1 biomarkers were most upregulated in I-BET151-treated parasites (Fig. 4). Several families of genes defined by Vigneron et al. (31) showed similar expression patterns at the early metacyclic stage and in I-BET151-treated parasites, including a set of 11 RBPs, the Fam50 genes (65) (Tb927.7.360, Tb927.7.400, Tb927.7.420, Tb927.7.440, and Tb927.7.380), the LPP genes Tb927.10.13400 and Tb927.10.13700, and the calpain genes (Tb927.11.1110, Tb927.1.2100, and Tb927.1.2120). Interestingly, SGE1, a member of the Fam50 gene family, has been shown to be upregulated in epimastigotes but downregulated thereafter (31). We found that SGE1 and other Fam50 family members in I-BET151-treated parasites are transiently upregulated at early time points and then downregulated thereafter (Fig. 4). However, the set of Fam10 salivary gland metacyclic (SGM) proteins that are most upregulated at the meta 1 and meta 2 stages of development are not upregulated in I-BET151-treated parasites. This supports the idea that I-BET151 treatment might initiate some features of the insect-stage developmental program but that other features are under the control of other gene-regulatory forces. SGM 1.7 has been definitively localized to the cell surface, and it is possible that surface protein remodeling requires additional checkpoints that are not initiated with I-BET151 treatment, as discussed above. Interestingly, the set of cluster 3 genes that peaks at 6 h in the I-BET151 data set (Fig. 6) is enriched in microtubule-based processes, and this is also a GO term set that is enriched in the early meta 1 parasites (31).

Independent of the effect of I-BET151 treatment on developmental genes, it is clear from the cluster analysis that I-BET151 treatment results in large global changes in gene expression and that these changes include both upregulation and downregulation (Fig. 5). In addition, the timing and pattern of the expression changes vary from early changes at 3 h that rebound quickly (clusters 1 and 2) to slower changes that are more sustained (clusters 3 and 4). Given the observation that large numbers of genes show similar expression patterns, we favor a model wherein I-BET151 treatment influences the expression of one or a few RNA-binding proteins that themselves regulate large numbers of genes through interaction with their 3′ untranslated regions (UTRs) (66, 67). RNA-binding proteins have been shown to be critical for postranscriptional regulation in trypanosomes (reviewed in reference 68) and have also been implicated in differentiation processes in both bloodstream (69–72) and insect-stage (73–76) forms. Indeed, we find that 8 of the 11 RBPs reported by Vigneron et al. to be differentially expressed during insect stage development were differentially expressed in our data set, including RBP10 (Tb927.8.2780), which is upregulated 2-fold after I-BET151 treatment and also upregulated at early metacyclic stages (31). One reason why some features of insect-stage differentiation, such as surface protein expression, may not be recapitulated in I-BET151-treated parasites is that one or more RBPs important for this process are not activated following drug treatment. This would be consistent with a model wherein different RBPs may be responsible for different aspects of the differentiation program, and thus, it may be possible to induce some parts of the program and not others.

While both TbBdf2 and TbBdf3 have been shown to bind I-BET151 *in vitro* (23), it remains unknown whether this molecule can bind any of the other five predicted bromodomain proteins in T. brucei. Elegant work by Staneva et al. showed that while 6 of the 7 predicted trypanosome bromodomain proteins localize to transcription start sites, these proteins do not exist together in a single complex (17). Thus, trypanosome bromodomain proteins likely have redundant but not completely overlapping functions, and it would be interesting to ascertain if other trypanosome bromodomain proteins bind to I-BET151. While many of the effects of I-BET151 in bloodstream parasites can be recapitulated by individual knockdown of TbBdf3, it remains possible that I-BET151 could have off-target effects. Future studies could address this possibility by identifying I-BET151-bound molecules in trypanosome lysate using mass spectrometry. Future work could also address whether perturbation of individual RNA binding proteins that are altered following I-BET151 treatment in insect-stage parasites might recapitulate portions of the differentiation program. More broadly, an increased understanding of gene-regulatory mechanisms in this highly diverged eukaryote may shed light on how gene-regulatory systems evolved across diverse forms of life.

## MATERIALS AND METHODS

### Parasite culture and drug treatment.

Procyclic PF427 parasites were cultured in SDM79 medium supplemented with 10% fetal calf serum (FCS) at 27°C. Parasites were treated with 20 μM I-BET151 (Sigma-Aldrich). Parasites were counted every 48 h and diluted to a concentration of 2 million/mL in fresh medium with fresh drug every 2 days. *EP1*/*GFP* procyclic reporter parasites were generated as described in reference 30. *EP1*/*GFP* bloodstream parasites (30) and single-marker (SM) parasites (77) (used as controls for verification of the *EP1*/*GFP* reporter construct) were grown in HMI9 supplemented with 10% FCS and 10% Serum Plus at 37°C and 5.0% CO_2_.

### PCR primers.

PCR was used to confirm correct integration of the *EP1*/*GFP* reporter construct using a primer upstream of *EP1* (GTCCGATAGGTATCTCTTATTAGTATAG) and within *GFP* (AGAAGTCGTGCTGCTTCATGTGGT). Primers specific to a region downstream of *EP1* were used as controls (GGCCATACTAGTCTTTGAATTTGGATCTTAAAATTATTATTG and GGCCATCTCGAGCAACTTCAGCTGCGGGGC).

### Library preparation and sequencing.

RNA extractions were performed using RNA Stat-60 (Tel-Test) according to the manufacturer’s instructions. Five micrograms of RNA was subjected to DNase treatment and poly(A)^+^ selection using the NEBNext poly(A) mRNA magnetic isolation module (E7490). High-throughput sequencing libraries were generated using the NEBNext Ultra directional RNA library preparation kit (E7420). Sequencing was performed on an Illumina HiSeq 2000 sequencer using 100-bp reads.

### Bioinformatic analysis.

Trimming for quality was performed on fastq files using TrimGalore from Babraham Bioinformatics (http://www.bioinformatics.babraham.ac.uk/projects/trim_galore/) with level 3 stringency using the command trim_galore—stringency 3. Trimmed reads were aligned uniquely to the Tb927v5 reference genome using Bowtie, allowing 2 mismatches with the command bowtie—best—strata -t -v 2 -a -m 1. For the VSG analysis, reads were aligned to the 427 VSGnome (7) using the same parameters. Raw counts and reads per kilobase per million (RPKM) for reads assigned to each gene were calculated using SeqMonk from Babraham Bioinformatics (https://www.bioinformatics.babraham.ac.uk/projects/seqmonk/). The DESeq2 (78) R package was used to identify differentially expressed genes and calculate Benjamini-Hochberg-adjusted *P* values. The Mfuzz R package (32) was used to cluster differentially expressed genes with a Benjamini-Hochberg-adjusted *P* value of less than 0.01 using a likelihood ratio test (LRT) analysis. Cluster members with a membership score greater than 0.7 (high core members) are provided in Table S7.

GSEA (79) was used to identify gene sets that were overrepresented in the set of differentially expressed genes with the following flags: -collapse false -mode Max_probe -norm meandiv -nperm 1000 -permute gene_set -rnd_type no_balance -scoring_scheme weighted -rpt_label and -sort real -order descending -create_gcts false -create_svgs false -include_only_symbols true -make_sets true -median false -num 100 -plot_top_x 20 -rnd_seed timestamp -save_rnd_lists false -set_max 500 -set_min 3 -zip_report false.

Gene sets are provided in Table S1. Normalized read counts and *P* values for differential expression are provided in Tables S2 and S3 and in Table S4, respectively.

GO analysis on each cluster was performed using TriTrypDB. GO results for cluster 3 are provided in Table S5.

### Flow cytometry.

Flow cytometry on *EP1*/*GFP* reporter parasites was performed on a Novocyte 2000R from Agilent (formerly Acea Biosciences) and analyzed using FlowJo software.

### Data availability.

Fastq files for RNA-seq are available from the NCBI SRA under accession number PRJNA924324.
