# Supplementary material for: Chemical Inhibition of Bromodomain Proteins in Insect-Stage African Trypanosomes Perturbs Silencing of the Variant Surface Glycoprotein Repertoire and Results in Widespread Changes in the Transcriptome
Source: Microbiol Spectr. 2023 Apr 25;11(3):e00147-23. doi: 10.1128/spectrum.00147-23 (PMC10269879; doi:10.1128/spectrum.00147-23)
Supplement: Supplemental file 1 — Supplemental material. Download spectrum.00147-23-s0001.pdf, PDF file, 3.0 MB [file spectrum.00147-23-s0001.pdf]

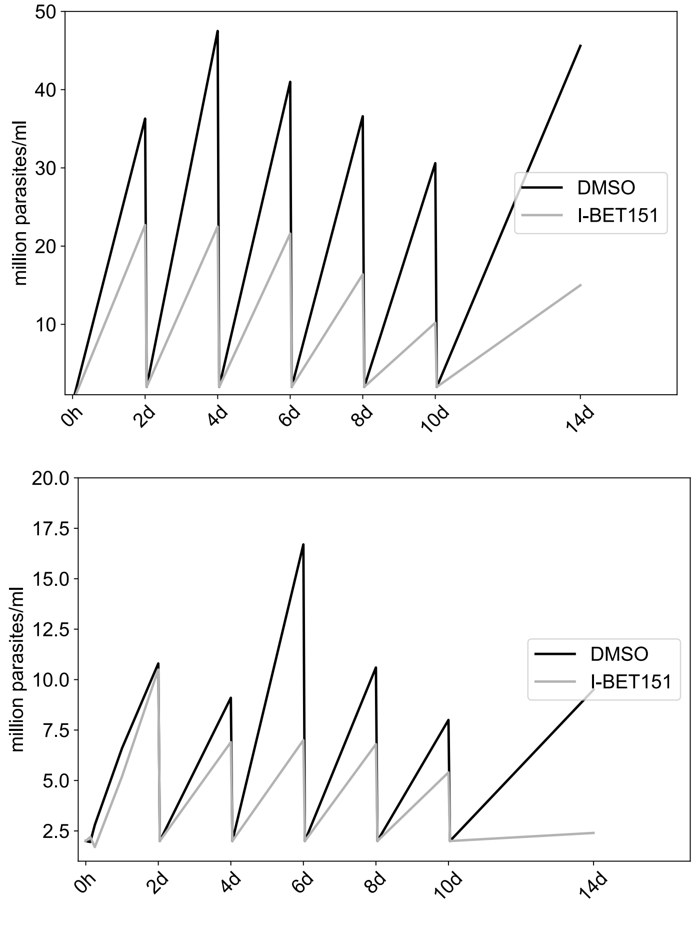

Supplemental Figure 1. Growth data for parasites grown with DMSO (control) or I-BET151 for two different experiments. Parasites were passaged every 2 days down to a concentration of 2 million/ml and counts were taken every 48 hours. Top panel parasites were harvested for RNA-seq.

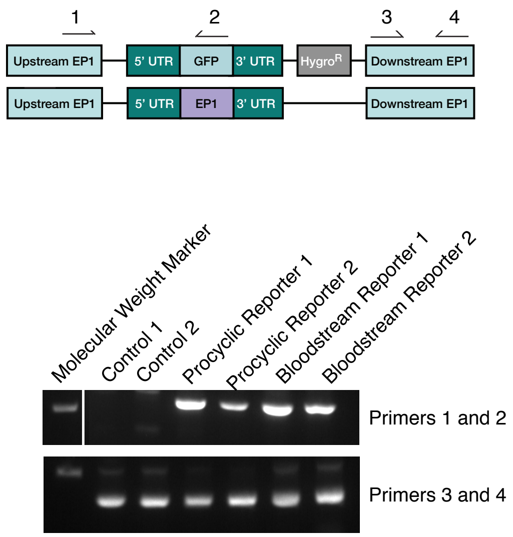

Supplemental Figure 2. Top, schematic for *EP1/GFP* reporter construct integrated at the *EP1* locus in procyclic stage parasites. Bottom, PCR confirmation of correct integration of the *EP1/GFP* reporter construct. Genomic DNA was isolated from indicated parasite lines and PCR was performed with primers specific to correct integration (Primers 1 and 2) and control primers (Primers 3 and 4). Control 1 and Control 2 are samples from the Single Marker (SM) bloodstream parasite line that does not contain the *EP1/GFP* reporter construct.

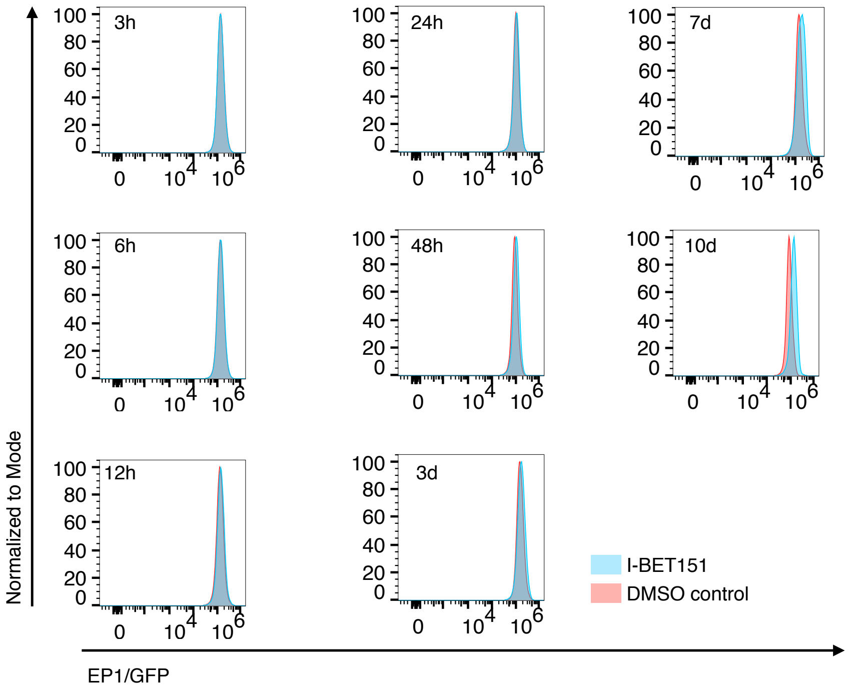

Supplemental Figure 3. Flow cytometry for *EP1/GFP* expression in I-BET151 treated parasites for the indicated length of time and compared to a DMSO control.

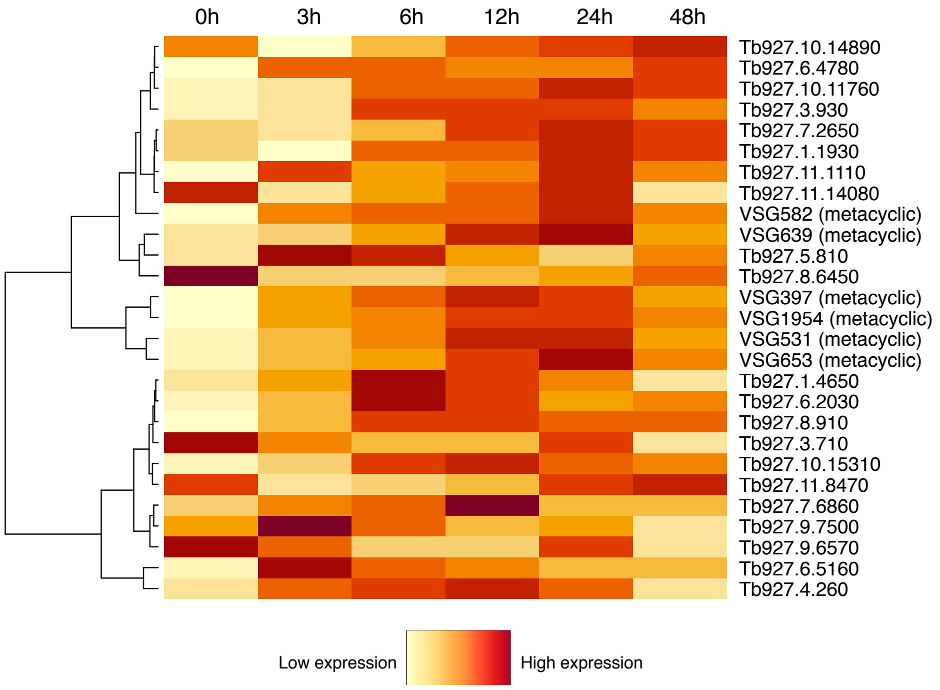

Supplementary Figure 4. Heatmap of early metacyclic Meta1 genes as described by Vigneron et al. for procyclic parasite RNA-seq samples at the indicated time of I-BET151 treatment.

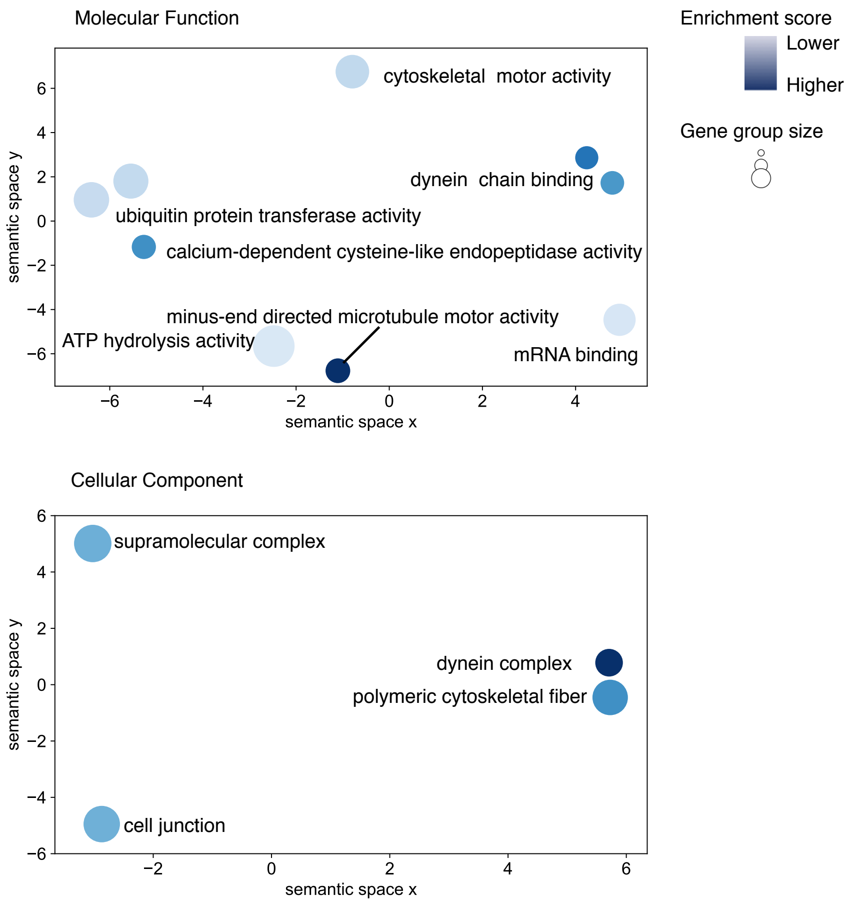

Supplemental Figure 5. REVIGO plot of GO enrichment analysis performed on Cluster 3 from the I-BET151 treated dataset.

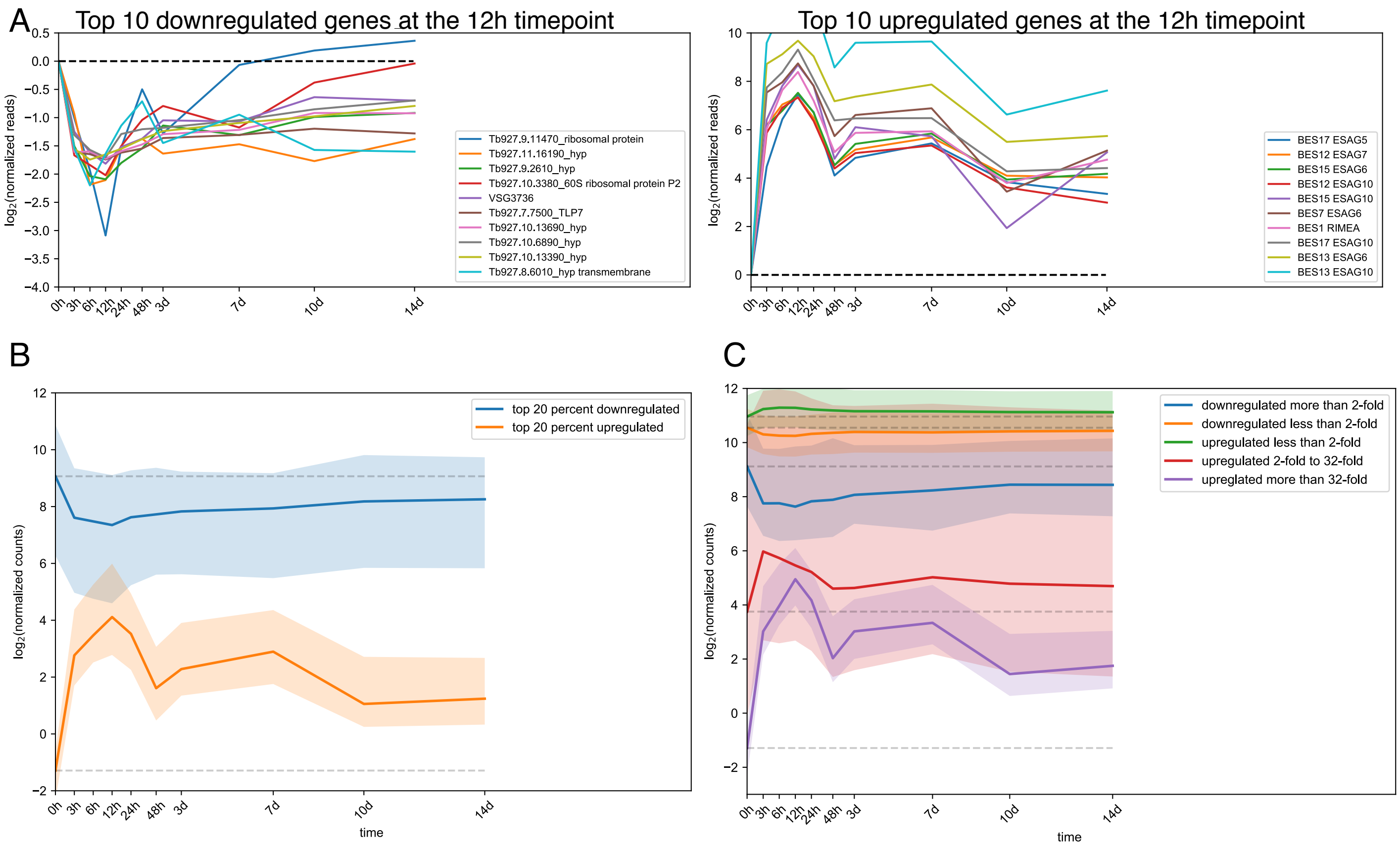

Supplemental Figure 6. Prolonged treatment with I-BET151 results in transcriptional adaptation. A) Normalized gene expression for the top 10 mostly highly upregulated or downregulated genes as measured by fold change at 12h of I-BET151 treatment compared to 0h. Expression level of each gene is shown over a period of 14 days. Genes were scaled to have a starting expression value of 1. Dashed line indicates the starting value of expression for each gene. **B)** Median normalized expression levels for the top 20% of downregulated or upregulated genes as measured by fold change after 12h of I-BET151 treatment compared to 0h treatment. Expression level of each gene is shown over a period of 14 days. Shading indicates the inner quartile range of expression for each group of genes. Dashed line indicates the starting median value of expression for each gene group. **C)** Same as in B except this time genes are separated into 5 groups based on their fold change value at 12h of I-BET151 treatment compared to 0h. Dashed line indicates the starting median value of expression for each gene group.

Table S1. Gene set enrichment results
Table S2. RPKM normalized counts for RNA-seq analysis.
Table S3. DESeq normalized counts for RNA-seq analysis.
Table S4. p-values for DESeq determined differentially expressed genes
Table S5. GO results for Cluster 3
Table S6. Bloodstream vs Procyclic upregulated and downregulated genes following I-BET151 treatment
Table S7. Cluster membership for genes with a membership score > 0.7
